# Ventilatory response during exercise among chronic Chagas cardiopathy patients

**DOI:** 10.1590/S1516-31802006000500010

**Published:** 2006-09-07

**Authors:** Fátima Palha de Oliveira, Roberto Coury Pedrosa

**Keywords:** Chagas disease, Oxygen consumption, Ventilation, Carbon dioxide, Exercise test, Doença de Chagas, Consumo de oxigênio, Ventilação, Dióxido de carbono, Teste de esforço

## Abstract

**CONTEXT AND OBJECTIVE:**

The change in slope of the $\dot{V}\text{E}/\dot{V}{\text{CO}}_{2}$ curve with time during exercise ($\dot{V}\text{E}/\dot{V}{\text{CO}}_{2}$ slope) has been recommended as a parameter for analyzing the ventilatory response during exercise among patients with heart failure of different etiologies. The aim of this work was to evaluate the ventilatory response among patients with chronic Chagas cardiopathy.

**METHODS:**

Forty-eight patients, divided into four groups according to the Los Andes clinical/hemodynamic classification, were studied. They were also classified according to peak oxygen uptake (peak $\dot{V}{\text{O}}_{2}$) for a second analysis. The results from the patients were compared with results from a control group consisting of 21 healthy male volunteers (no Chagas disease). Exercise was performed on a cycle ergometer with loads increasing at the rate of 12.5 watts/min, and exercise duration was symptom-limited. Gas concentration and flow rate data were fed into a computer, which produced a real-time report on ventilatory and gas exchange parameters. (breath-by-breath). The ventilatory parameters of $\dot{V}\text{E}/\dot{V}{\text{CO}}_{2}$ slope and $\dot{V}\text{E}/\dot{V}{\text{CO}}_{2}$ ratio computed at different times of the test were adopted.

**RESULTS:**

Although there were no significant differences in $\dot{V}\text{E}/\dot{V}{\text{CO}}_{2}$ ratio and $\dot{V}\text{E}/\dot{V}{\text{CO}}_{2}$ slope when patients were grouped using the Los Andes clinical/hemodynamic classification, these parameters varied significantly when peak $\dot{V}{\text{O}}_{2}$ was used to define patient groups.

**CONCLUSION:**

Our results indicate that progressive deterioration in ventilatory response among chronic Chagas cardiopathy patients during exercise is more evident when the functional capacity (peak $\dot{V}{\text{O}}_{2}$) is reduced, than when changes are related to the Los Andes classification.

## RESUMO

**CONTEXTO E OBJETIVO:**

Mudança na inclinação da curva $\dot{V}\text{E}/\dot{V}{\text{CO}}_{2}$ ao longo do tempo durante exercício tem sido recomendada como parâmetro de análise da resposta ventilatória durante o exercício entre pacientes com insuficiência respiratória de diferentes etiologias. O objetivo deste estudo foi avaliar a resposta ventilatória de pacientes com cardiopatia chagásica crônica.

**MÉTODOS:**

Total de 48 pacientes, divididos em quatro grupos de acordo com a Classificação Clinica e Hemodinâmica de Los Andes, compôs a amostra deste estudo. Na análise, os pacientes foram classificados também pelo consumo de oxigênio obtido em teste de esforço. Os resultados dos pacientes foram comparados com os resultados do grupo de controle, formado por 21 homens saudáveis, sem diagnóstico de doença de Chagas. O teste de esforço foi realizado em bicicleta ergométrica com o incremento de 12.5 W/min, sendo a duração do exercício sintoma-limitado. A concentração de gases e o fluxo ventilatório foram medidos em tempo real e registrados ciclo a ciclo. Foram adotados os parâmetros inclinação da curva de $\dot{V}\text{E}/\dot{V}{\text{CO}}_{2}$ e razão entre $\dot{V}\text{E}/\dot{V}{\text{CO}}_{2}$ (computada em diferentes momentos do teste).

**RESULTADOS:**

Apesar de não haver diferenças significativas para $\dot{V}\text{E}/\dot{V}{\text{CO}}_{2}$ *slope* e para a razão $\dot{V}\text{E}/\dot{V}{\text{CO}}_{2}$ quando os pacientes foram grupados pela Classificação Clínica/Hemodinâmica de Los Andes, esses parâmetros variaram significativamente quando a capacidade funcional, $\dot{V}{\text{O}}_{2}$ *peak*, foi considerada.

**CONCLUSÕES:**

Nossos resultados indicam que uma menor capacidade ventilatória durante o exercício foi mais evidente nos pacientes com cardiopatia chagásica crônica quando a capacidade funcional estava reduzida.

## INTRODUCTION

The change in slope of the $\dot{V}\text{E}/\dot{V}{\text{CO}}_{2}$ curve with time during exercise ($\dot{V}\text{E}/\dot{V}{\text{CO}}_{2}$ slope) has been recommended as a parameter for analyzing the ventilatory response during exercise among patients with heart failure of different etiologies.^1^

## OBJECTIVE

The aim of the present study was to evaluate ventilatory response among chronic Chagas cardiopathy (CCC) patients.

## MATERIALS AND METHODS

This study was a cross-sectional and descriptive analysis of 48 male patients with CCC (mean age: 51 ± 11 years). These patients were living in Rio de Janeiro and were not taking part in any systematic physical training program. All of these patients had been clinically stable over the three-month period preceding the study. They presented positive indirect hemagglutination reaction and indirect immunofluorescence for *Trypanosoma cruzi*, and no other associated disease. They were grouped according to the Los Andes clinical/hemodynamic classification,^2^ and the diagnosis of congestive heart failure met the criteria of the Framingham Heart Study.^3^

Selection of the patients for the study was performed among the 200 patients followed up in Hospital Universitário Clementino Fraga Filho, Universidade Federal do Rio de Janeiro, over the period from 1999 to 2000, and the data were analyzed in 2002. The exclusion criteria were: systemic arterial hypertension, chronic obstructive pulmonary disease, cardiomyopathy of any other type or cause, thyroid dysfunction, known immunological dysfunction, rheumatic valve disease, congenital heart diseases, obstructive coronary artery disease, use of heart pacemaker, neuro-muscular disorders, practicing of sports, and inability to reach the anaerobic threshold when undergoing the test.

The patients were grouped in two ways: firstly, using the Los Andes clinical/hemodynamic classification^2^; secondly, to determine whether alterations in the functional capacity of the cardiovascular system were associated with the ventilatory response to exercise, the patients were redistributed into four groups according to their peak $\dot{V}{\text{O}}_{2}$ independent of their degree of heart failure.

The exercise test was performed on a mechanically braked cycle ergometer (Monarch) and the patients using medications were instructed to suspend their use 48 hours prior to the test.

The exercise was begun with two minutes of warm-up (zero Watts). The workload was increased at a rate of 12.5 watts/min, with 60 rotations per minute, until the appearance of limiting symptoms. All of the patients and also a control group consisting of 21 healthy male volunteers performed the same test protocol.

The reproducibility of the exercise test in our laboratory was verified by means of test and retest of ten volunteers and there were no significant differences (paired and simple Student's t test, p < 0.05) between the results.

A rapid gas analyzer (Airspec MGA 2000) was utilized on a breath-by-breath basis, with a Fleisch pneumotachograph and a differential pressure transducer (Microswitch 163PC01D36). Equipment calibration was performed on a daily basis before each test.

We analyzed $\dot{V}\text{E}/\dot{V}{\text{CO}}_{2}$ (liter/min) by considering the average from the last five cycles at six different times: (a) at rest; (b) when $\dot{V}{\text{CO}}_{2}$ reached 0.5 liter/min; (c) when $\dot{V}{\text{CO}}_{2}$ reached 1.0 liter/min; (d) at the exercise peak; (e) after the first minute of recovery; and (f) after the third minute of recovery. The $\dot{V}\text{E}/\dot{V}{\text{CO}}_{2}$ slope during exercise period was also analyzed (linear regression analysis).^4^ The results from the patients were compared with the results from the 21 healthy male volunteers.

The local Ethics Committee approved this study and the patients and the control group underwent the tests only after providing written informed consent.

The statistical treatment (Pearson's correlation coefficient, one-way analysis of variance, ANOVA, post-hoc Tukey, HSD) were performed using the Statistical Analysis System (SAS), considering significant values of p < 0.05.

## RESULTS

**Figure 1:**
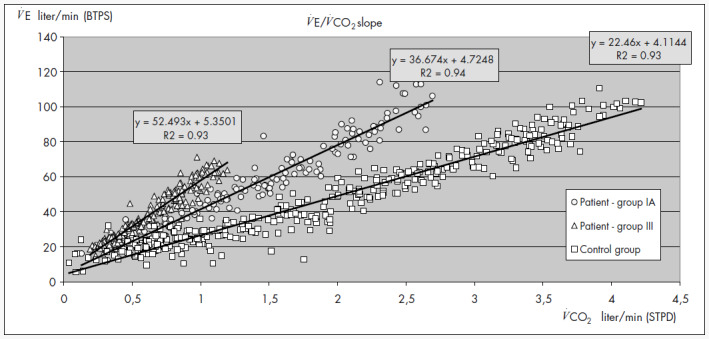
$\dot{V}\text{E/}\dot{V}{\text{CO}}_{\text{2}}$ slope during exercise (two patients and one control). BTPS = body temperature and pressure saturated; STPD = standard temperature and pressure, dry.

The functional capacity of patients in the initial phase (IA) of CCC was superior to that of patients in an advanced phase (group III) (Table 1, Figure 1). There was great variability in the peak $\dot{V}{\text{O}}_{2}$ in CCC (Table 2), and $\dot{V}\text{E}/\dot{V}{\text{CO}}_{2}$ and $\dot{V}\text{E}/\dot{V}{\text{CO}}_{2}$ slope had significant negative but weak correlations with peak $\dot{V}{\text{O}}_{2}$ and ventilatory anaerobic threshold (Table 3). $\dot{V}\text{E}/\dot{V}{\text{CO}}_{2}$ exhibited a tendency to decrease with effort and to return to resting values after three minutes of recovery. When patients were grouped according to peak $\dot{V}{\text{O}}_{2}$ (Table 4), there were significant differences in $\dot{V}\text{E}/\dot{V}{\text{CO}}_{2}$ and $\dot{V}\text{E}/\dot{V}{\text{CO}}_{2}$ slope between some groups of patients and controls.

**Table 1:** Mean results (mean ± standard deviation) for all variables measured in Chagas patients, grouped according to the Los Andes clinical/hemodynamic classification^2^

Ergospirometric variables	Control (n = 21)	Groups according to Los Andes Classification				ANOVA	Tukey
		IA (n = 16)	IB (n = 08)	II (n = 12)	III (n = 12)		
	No disease	Normal electrocardiogram and echocardiogram (no heart involvement)	Normal electrocardiogram and abnormal echocardiogram (mild heart involvement)	Abnormal electrocardiogram and echocardiogram, without congestive heart failure (advanced heart involvement)	Abnormal electrocardiogram and echocardiogram with congestive heart failure (severe heart involvement)	p	
**Age (years)**	**39**	**46**	**48**	**53**	**58**	0.0001	**C**≠ **II, III, III**≠ **IA**
	± 10	± 10	± 15	± 10	± 9		
**Body mass (kg)**	**74**	**79**	**66**	**66**	**71**	0.019	**IA**≠ **II**
	± 10	± 10	± 13	± 12	± 15		
**Height (m)**	**1.73**	**1.69**	**1.66**	**1.66**	**1.71**	0.032	**C**≠ **II**
	± 6	± 6	± 6	± 7	± 10		
**Peak** $\dot{V}{\text{O}}_{2}$ **(ml/min)**	**2418**	**1956**	**1542**	**1687**	**1273**	0.00002	**C**≠ **IB, II, III. IA**≠ **III**
	± 749	± 364	± 602	± 582	± 393		
**Peak** $\dot{V}{\text{O}}_{2}$ **(ml/kg/min)**	**33**	**25**	**23**	**26**	**19**	0.001	**C**≠ **IA, III**
	± 12	±5	±7	±9	±5		
**AT (ml O_2_/min)**	**1817**	**1608**	**1425**	**1251**	**1053**	0.005	**C**≠ **II, III**
	± 767	± 271	± 631	± 386	± 340		
$\dot{V}\text{E/}\dot{V}{\text{CO}}_{2}$ **at rest**	**58**	**58**	**66**	**61**	**63**	0.698	**ns**
	± 14	± 17	± 11	± 18	± 18		
$\dot{V}\text{E/}\dot{V}{\text{CO}}_{2}$ **at 0.5 liter CO_2_/min**	**43**	**44**	**50**	**46**	**51**	0.121	**ns**
	± 8	± 9	± 8	± 8	± 12		
$\dot{V}\text{E/}\dot{V}{\text{CO}}_{2}$ **at 1.0 liter CO_2_/min**	**36**	**38**	**42**	**38**	**41**	**0.308**	**ns**
	± 8	± 6	± 5	± 8	± 12		
$\dot{V}\text{E/}\dot{V}{\text{CO}}_{2}$ **at exercise peak**	**45**	**39**	**48**	**39**	**47**	**0.233**	**ns**
	± 13	± 9	± 13	± 10	± 18		
$\dot{V}\text{E/}\dot{V}{\text{CO}}_{2}$ **after 1 min of recovery**	**47**	**41**	**46**	**40**	**45**	**0.493**	**ns**
	± 15	± 8	± 12	± 12	± 16		
$\dot{V}\text{E/}\dot{V}{\text{CO}}_{2}$ **after 3 min of recovery**	**54**	**51**	**59**	**45**	**49**	**0.235**	**ns**
	± 16	± 15	± 14	± 12	± 15		
**$\dot{V}\text{E/}\dot{V}{\text{CO}}_{2}$ slope**	**37**	**33**	**39**	**31**	**38**	**0.394**	**ns**
	± 9	± 9	± 11	± 8	± 17		

C = control group; Peak $\dot{V}O_{2}$ = oxygen uptake in the last seconds of exercise; $\dot{V}E\text{/}\dot{V}CO_{2}$ = ventilation/carbon dioxide production ratio; AT = ventilatory anaerobic threshold; ANOVA = analysis of variance; ns: non-significant.

**Table 2:** Distribution of Chagas patients among Los Andes groups versus classification by $\dot{V}{\text{O}}_{\text{2}}$ attained at exercise peak

Los Andes groups	$\dot{V}{\text{O}}_{\text{2}}<16$	16 <$\dot{V}{\text{O}}_{\text{2}}$< 20	20 < $\dot{V}{\text{O}}_{\text{2}}$ < 29	$\dot{V}{\text{O}}_{\text{2}}>29$	Total
**IA**	0	4	9	3	16
**IB**	1	3	2	2	8
**II**	1	2	7	2	12
**III**	3	2	5	1	12
**Total**	**5**	**11**	**23**	**8**	**47**

$\dot{V}O_{2}$ = oxygen uptake (ml/kg/min).

**Table 3:** Correlation coefficient of $\dot{V}\text{E}/\dot{V}{\text{CO}}_{\text{2}}$ with peak $\dot{V}{\text{O}}_{\text{2}}$ and ventilatory anaerobic threshold of Chagas patients

	$\dot{V}\text{E}/\dot{V}{\text{CO}}_{\text{2}}$						$\dot{V}\text{E}/\dot{V}{\text{CO}}_{\text{2}}$ slope
	Rest	0.5 liter CO_2_/min	1.0 liter CO_2_/min	Exercise peak	After 1 min of recovery	After 3 min of recovery	
**Peak $\dot{V}{\text{O}}_{\text{2}}$ (ml/min)**	- 0.41	- 0.45	- 0.47	- 0.38	- 0.37	- 0.30	- 0.28
	p = 0.001	p = 0.000	p = 0.000	p = 0.001	p = 0.002	p = 0.013	p = 0.020
**Peak $\dot{V}{\text{O}}_{\text{2}}$ (ml/kg/min)**	- 0.29	- 0.43	- 0.45	- 0.36	- 0.30	- 0.28	- 0.33
	p = 0.018	p = 0.001	p = 0.000	p = 0.003	p = 0.014	p = 0.023	p = 0.007
**AT (ml/min)**	- 0.36	- 0.34	- 0.32	- 0.27	- 0.28	- 0.15	- 0.12
	p = 0.004	p = 0.007	p = 0.009	p = 0.031	p = 0.023	**p = 0.253**	**p = 0.331**

$\dot{V}O_{2}$ = oxygen uptake; $\dot{V}E/\dot{V}CO_{2}$ *= ventilation/carbon dioxide production; AT = ventilatory anaerobic threshold.*

**Table 4:** Mean results (mean ± standard deviation) for all variables measured in Chagas patients, grouped according to peak $\dot{V}{\text{O}}_{\text{2}}$

Groups	Control (n = 18)	$\dot{V}{\text{O}}_{\text{2}}<16$ ml/kg/m (n = 05)	16 <$\dot{V}{\text{O}}_{\text{2}}$ < 20ml/kg/m (n = 11)	20<$\dot{V}{\text{O}}_{\text{2}}$ <29 ml/kg/m (n = 23)	$\dot{V}{\text{O}}_{\text{2}}$ >29 ml/kg/m (n = 08)	ANOVA ^p^	Tukey
**Age (years)**	41 ± 9	62 ± 7	54 ± 10	51 ± 9	37 ± 11	0.000003	C ≠1, 2, 3 4 ≠1, 2, 3
**Body mass (kg)**	74 ± 11	64 ± 14	69 ± 13	75 ± 13	65 ± 7	0.120	non significant
**Height (m)**	173 ± 6	165 ± 8	163 ± 5	170 ± 5	167 ± 6	0.0005	C, 3, ≠ 2
**Peak $\dot{V}{\text{O}}_{\text{2}}$ (ml/min)**	2592 ± 660	838 ± 233	1259 ± 242	1836 ± 333	2253 ± 392	0.000001	C ≠ 1, 2, 3 4 ≠ 1, 2 3 ≠ 1, 2
**Peak $\dot{V}{\text{O}}_{\text{2}}$ (ml/kg/min)**	36 ± 11	13 ± 2	18 ± 1	25 ± 2	35 ± 5	0.000001	C ≠ 1, 2, 3 4 ≠ 1, 2, 3 3 ≠ 1
**AT (ml/min)**	1942 ± 773	768 ± 147	1062 ± 239	1504 ± 335	1690 ± 506	0.00004	C ≠ 1, 2 4 ≠ 1
$\dot{V}\text{E}/\dot{V}{\text{CO}}_{\text{2}}$ **at rest**	57 ± 15	72 ± 9	71 ± 11	57 ± 17	54 ± 17	0.025	1 ≠ 3, 4 2 ≠ 3, 4, C
$\dot{V}\text{E}/\dot{V}{\text{CO}}_{\text{2}}$ **at 0.5 liter CO_2_/min**	42 ± 7	54 ± 11	53 ± 8	45 ± 9	42 ± 11	0.006	C ≠2
$\dot{V}\text{E}/\dot{V}{\text{CO}}_{\text{2}}$ **at 1.0 liter CO_2_/min**	36 ± 9	43 ± 4	45 ± 8	38 ± 7	34 ± 5	0.005	C, 4 ≠ 2
$\dot{V}\text{E}/\dot{V}{\text{CO}}_{\text{2}}$ **at exercise peak**	45 ± 14	60 ± 19	50 ± 9	39 ± 9	33 ± 4	0.0001	1 ≠ 3, 4 2 ≠ 3, 4
$\dot{V}\text{E}/\dot{V}{\text{CO}}_{\text{2}}$ **after 1 min of recovery**	45 ± 15	53 ± 15	50 ± 10	40 ± 10	33 ± 5	0.008	4 ≠ 1, 2
*$\dot{V}\text{E}/\dot{V}{\text{CO}}_{\text{2}}$* **after 3 min of recovery**	52 ± 16	63 ± 14	58 ± 14	46 ± 12	45 ± 16	0.047	non significant
*$\dot{V}\text{E}/\dot{V}{\text{CO}}_{\text{2}}$* **slope**	37 ± 10	47 ± 18	41 ± 9	32 ± 9	27 ± 6	0.002	1 ≠ 3, 4 2 ≠ 4

Groups: C = control (peak $\dot{V}O_{2}$ > 22 ml/kg/min); Peak $\dot{V}O_{2}$ = oxygen uptake in the last seconds of exercise; $\dot{V}E/\dot{V}CO_{2}$ = ventilation/Carbon dioxide production ratio; AT = ventilatory anaerobic threshold; ANOVA = analysis of variance.

## DISCUSSION

This study demonstrated an inverse relationship between the degree of heart disease in CCC and the patients' functional capacity (peak $\dot{V}{\text{O}}_{\text{2}}$ and anaerobic threshold, Table 3). Both controls and patients in the initial phase of the disease (group IA) were more tolerant of exercise than were patients in advanced stages of CCC (Table 1).

No significant differences in ventilatory efficiency could be seen between the patient groups organized according to the Los Andes classification (Table 1). This contrasted with the classification according to functional capacity (Table 4). Results of this nature have also been found via another mathematical method that considers ventilation and functional capacity.^5^ The variations in peak $\dot{V}{\text{O}}_{\text{2}}$ values (Table 2) among patients with the same Los Andes classification who are characterized clinically as having severe heart involvement were probably due to the development of compensatory muscle adaptations. These muscle adaptations result in better exercise tolerance and reduce the impact of cardiopathy consequences. Certainly these patients were in a better condition: a few of the participants had $\dot{V}{\text{O}}_{\text{2}}>20$ ml/kg/min. Analysis of $\dot{V}\text{E}/\dot{V}{\text{CO}}_{\text{2}}$ and $\dot{V}\text{E}/\dot{V}{\text{CO}}_{\text{2}}$ slope is a practical method that does not need maximum effort for differentiation of patients' functional capacities. Thus, this becomes another index for classifying patients who are waiting for heart transplantation.

Greater peak $\dot{V}{\text{O}}_{\text{2}}$ and smaller $\dot{V}\text{E}/\dot{V}{\text{CO}}_{\text{2}}$ slope reflect ventilatory efficiency throughout the exercise period (Table 4). Chagas patients with reduced functional capacity exhibited greater $\dot{V}\text{E}/\dot{V}{\text{CO}}_{\text{2}}$ slope, thus reproducing the results obtained from non-Chagas cardiopa-thies.^1^ These results indicate that the patients needed greater ventilatory effort for the equivalent quantity of CO_2_ produced during exercise than did individuals with better functional capacity (Figure 1).

Grouping patients according to peak $\dot{V}{\text{O}}_{\text{2}}$ also served to emphasize differences in ventilatory response at the beginning of the exercise ($\dot{V}\text{E}/\dot{V}{\text{CO}}_{\text{2}}$, at 0.5 liter/min of $\dot{V}{\text{CO}}_{\text{2}}$). At this exercise level, aerobic metabolism probably predominates, thereby eliminating any need for an altered ventilatory response that would compensate for an increase in lactic acid. A likely candidate for such alterations at this phase of the effort is the change in V_D_/V_T_ (fraction of the tidal volume that contributes to the dead space), which some authors have detected during exercise in patients with heart failure.^6^

The usefulness of $\dot{V}\text{E}/\dot{V}{\text{CO}}_{\text{2}}$ in predicting survival rates for patients with congestive heart failure has been demons trated:^1^ after 18 months, the survival rate was 95% for patients that attained $\dot{V}\text{E}/\dot{V}{\text{CO}}_{\text{2}}$ values of less than 34 at the exercise peak. Since patients with better tolerance of exercise, as represented by lower $\dot{V}\text{E}/\dot{V}{\text{CO}}_{\text{2}}$ ratios and higher $\dot{V}{\text{O}}_{\text{2}}$ values, tend to have better prognoses, the functional capacity classification may better quantify the disability caused by heart disease than does the Los Andes clinical/hemodynamic classification.

## CONCLUSIONS

Peak $\dot{V}{\text{O}}_{\text{2}}$, $\dot{V}\text{E}/\dot{V}{\text{CO}}_{\text{2}}$ and $\dot{V}\text{E}/\dot{V}{\text{CO}}_{\text{2}}$ slope were shown to be useful parameters for classifying individuals' cardiopulmonary conditions. The $\dot{V}\text{E}/\dot{V}{\text{CO}}_{\text{2}}$ parameters had the advantage of not requiring maximum levels of exercise.

Progressive deterioration in the ventilatory response among CCC patients during exercise was more evident when the functional capacity (peak $\dot{V}{\text{O}}_{\text{2}}$) was considered.
